# Extraction of average neck flexion angle during swallowing in neutral and chin-tuck positions

**DOI:** 10.1186/1475-925X-8-25

**Published:** 2009-10-07

**Authors:** Delbert Hung, Ervin Sejdić, Catriona M Steele, Tom Chau

**Affiliations:** 1 Bloorview Research Institute, Bloorview Kids Rehab, Toronto, Ontario, Canada; 2 Institute of Biomaterials and Biomedical Engineering, University of Toronto, Toronto, Ontario, Canada; 3 Toronto Rehabilitation Institute, Toronto, Ontario, Canada; 4 Department of Speech-Language Pathology, University of Toronto, Toronto, Ontario, Canada

## Abstract

**Background:**

A common but debated technique in the management of swallowing difficulties is the chin tuck swallow, where the neck is flexed forward prior to swallowing. Natural variations in chin tuck angles across individuals may contribute to the differential effectiveness of the technique.

**Methodology:**

To facilitate the study of chin tuck angle variations, we present a template tracking algorithm that automatically extracts neck angles from sagittal videos of individuals performing chin tuck swallows. Three yellow markers geometrically arranged on a pair of dark visors were used as tracking cues.

**Results:**

The algorithm was applied to data collected from 178 healthy participants during neutral and chin tuck position swallows. Our analyses revealed no major influences of body mass index and age on neck flexion angles during swallowing, while gender influenced the average neck angle only during wet swallows in the neutral position. Chin tuck angles seem to be independent of anthropometry and gender in healthy adults, but deserve further study in pathological populations.

**Conclusion:**

The proposed neck flexion angle extraction algorithm may be useful in future studies where strict participant compliance to swallowing task protocol can be assured.

## Introduction

Dysphagia is the umbrella term used to describe a large range of swallowing difficulties. Most of these difficulties arise from insults to motor or sensory pathways to the brain, which can be the result of congenital neurological conditions [1] or acute stroke [2]. In addition to neurogenic origins, dysphagia can also arise from anatomical abnormalities or physical damage to the structures involved in swallowing, for example, following tumor resectioning in the pharyngeal compartment [3].

Dysphagia in symptomatic patients almost always involves impairment of swallowing during the oral or pharyngeal phases [4]. Patients with oral phase dysphagia often complain of difficulties handling the bolus in the oral cavity, indicative of neurological deficiencies in voluntary control of the tongue or jaw. However, these patients often succeed in swallows if the pharyngeal stage can be triggered using compensatory maneuvers. The symptoms and causes of pharyngeal dysphagia are more varied due to the high involvement of involuntary constrictor muscles in the process. Food particles may be lodged in the pharyngeal recesses due to improper clearance caused by weakened constrictors or failure to open the upper esophageal sphincter. Patients may complain of frequent coughs during swallowing, accumulation of phlegm, or the sensation of foreign bodies lodged in their throat. The latter is more problematic since lingering food particles in the pharynx could be dislodged into the trachea (aspiration), increasing the likelihood of aspiration pneumonia [4]. In more severe cases where the patient has partially lost sensation in the pharynx, aspirations can occur silently without the cough or gag reflex. In either case, the presence of particles in the trachea is indicative of aspiration risk and accurate methods of aspiration detection are required to assess the severity of the patient's condition.

As mentioned, patients with dysphagia often aspirate during feeding (entry of ingested food or liquids into the larynx below the level of the true vocal folds) [5,6]. Common techniques used by speech-language pathologists to treat patients at risk of aspiration include the pre-processing of food and the variation of head angle during swallowing [5]. Amongst the latter is the chin-tuck swallow, where patients are instructed to tuck their chin towards their chest during each swallow. While commonly applied in clinical practice, efficacy of the chin-tuck swallow in preventing aspiration is debated in literature, with aspiration prevention rates at about 50% [5].

Previous studies investigating the source of variation of chin-tuck swallows have reported an anatomical link to efficacy of airway protection, but findings have been mixed. In particular, the epiglottic angle was significantly higher for patients where the chin-tuck was unsuccessful at mitigating aspiration [6]. A similar study noted that the chin-tuck maneuver elicited a posterior shift in anterior pharyngeal structures, which resulted in a narrowed laryngeal entrance, and is hypothesized to improve airway protection [7]. In contrast, a videomanometric study revealed that the chin-tuck position results in weaker pharyngeal contractions [8], which are linked to increased aspiration risk [9]. Clearly there are variations in the effect of the chin-tuck swallow with a close association to head position, particularly neck angle. Previous studies however have not quantified actual neck angles during the chin-tuck procedure.

In this paper, we thus study potential neck angle variations among people performing wet (i.e., in a neutral head position) and wet chin-tuck swallows and quantitatively gauge the association of neck angle with anthropometric and demographic variables. In so doing, we demonstrate a computer-vision based method of automatically extracting neck angles in the sagittal plane during different swallowing tasks. The extracted angles are then analyzed with respect to age, gender and body mass index.

## Methodology

### Data acquisition

**Figure 1:**
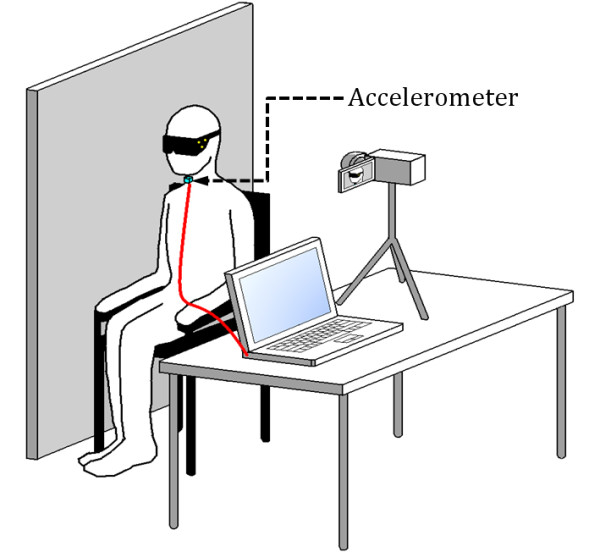
**Experimental setup**. The experimental setup used in the study.

**Figure 2:**
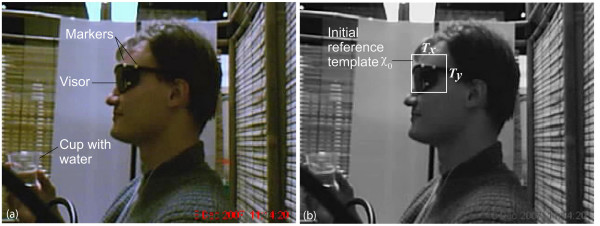
**Data acquisition approach and marking of the initial reference template**. The acquired data: (a) a participant wearing the standard-issue visor with markers; (b) the initial reference template for tracking head position.

In this study, four hundred and eight participants (aged 18-65) were recruited and all provided written consent. The study protocol was approved by the research ethics boards of the Toronto Rehabilitation Institute and Bloorview Kids Rehab, both located in Toronto, Ontario, Canada. Participants sat behind a screen for privacy and answered a set of questions relating to medical and swallowing history. A speech language pathologist measured the height, weight, body fat percentage (BIA Meter, BC-550, Tanita), neck circumference and mandibular jaw length of each participant. A webcam (DX-DTCAM, Dynex) mounted on a table next to the participant's chair was set up to capture a sagittal view of the participant's face and neck at a resolution of 320 × 240 pixels, at 15 frames per second, as shown in Figure 1. In addition, three yellow dots were positioned on the visor in a triangular pattern as shown in Figure 2. These markers were used to track the position of a participant's head. Furthermore, a dual-axis accelerometer (ADXL322, Analog Devices) was attached to the participant's neck (anterior to the cricoid cartilage) using double-sided tape. Data were collected using a custom LabVIEW program running on a laptop computer and saved for subsequent off-line analysis.

Each participant was cued to perform 5 saliva swallows, 5 water swallows by cup with their chin in the neutral head position (i.e., perpendicular to the floor) and 5 water swallows in the chin-tucked position (neck flexed). The entire data collection session lasted 15 minutes per participant. The webcam captured images only during wet swallows in the neutral and chin tuck positions. Via retrospective video review, we identified participants who did not strictly follow the experimental protocol (e.g., exhibited excessive out-of-plane movement). These data were eliminated from further consideration.

### Image processing algorithm for tracking of neck flexion angles

Let (*x, y*) with *x, y *∈ ℤ^+^, represent the row and column coordinates of an image, respectively, where (1,1) denotes the top-left pixel of an image. Let *z *∈ {1, 2, 3} denote the color component in red-green-blue (RGB) color space. Thus, we denote a raw *N *× *M *RGB image as *f*(*x, y, z*), with *f*(*x, y, z*) ∈ ℤ*, 1 ≤ *x *≤ *N *and 1 ≤ *y *≤ *M*. For example, R, G and B pixels have the forms *f*(*x, y*, 1), *f*(*x, y*, 2) and *f*(*x, y*, 3), respectively. Since we deal with 24-bit images (8-bits per color channel), 0 ≤ *f*(*x, y, z*) ≤ 255.

#### Template characterization

**Figure 3:**
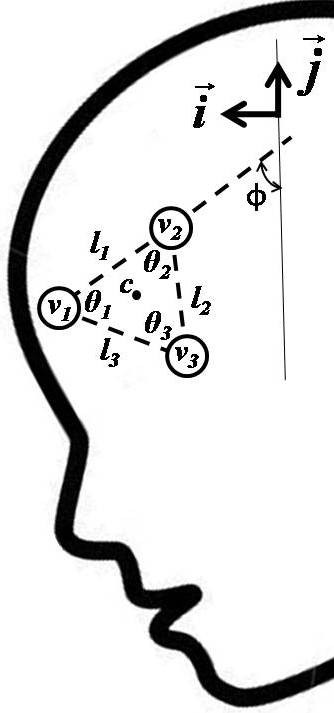
**Key geometrical quantities used for estimation of neck flexion angles**. Definition of key geometrical quantities for estimating neck flexion angle.

As shown in Figure 2(b), the initial reference template, *χ*_0_, is a sub-image manually cropped from a segmented grayscale version of the first frame of the video such that the markers are well contrasted against the dark visor background. The width and height of the template in pixels is *T*_*x *_and *T*_*y*_, respectively. The location of the markers in *χ*_0 _is then determined by selecting the three highest intensity pixels, which define the vertices **v**_**1**_, **v**_**2**_, **v**_**3 **_of a triangle, as depicted in Figure 3. Here, each vertex is a coordinate pair designating a row and column in the image, i.e.,**v**_**i **_= (, ), ∀*i*, with 1 ≤  ≤ *N *and 1 ≤  ≤ *M*. The vertices define three vectors, denoted as ,  and . To facilitate subsequent computations, we also define unit vectors,  and , in the horizontal and vertical directions of the image, respectively. The side lengths *l*_1_, *l*_2 _and *l*_3 _were estimated as the Euclidean distances between vertices while angles *θ*_1_, *θ*_2 _and *θ*_3 _between each pair of vectors were estimated using the dot product formula. Finally, we define the centroid of the vertices, **c **= (*c*^*x*^, *c*^*y*^), as .

The orientation angle, *ϕ*, is defined as:

(1)

where  is the vector defined by vertices **v**_**1 **_and **v**_**2 **_and  is the unit vector in the vertical direction. Equation (1) defines *ϕ *as the acute angle between the vertical axis of the image and the line extrapolated from the base of the triangular marker pattern as shown in Figure 3. The initial side lengths and angles derived from *χ*_0_, are denoted as {*L*_1_, *L*_2_, *L*_3_, Θ_1_, Θ_2_, Θ_3_} and characterize the "correct" reference marker set. In other words, when analyzing subsequent frames, candidate marker coordinates that closely resemble this reference set are more likely to be a correct match.

#### Segmentation

The segmentation phase of the algorithm nullified extraneous image background from each frame, according to colour and pixel intensity criteria. The markers of interest were yellow dots contrasted against a black visor background as shown in Figure 2(a). To automatically isolate these markers in subsequent frames, we derive two binary masks: one representing sets of pixels that are yellow and the other, pixels that contribute to a dark background.

To create a mask which isolates yellow dots, the colour information in the current frame is manipulated. Defining a set of rules of to isolate "yellow" pixels is problematic in the RGB colour model as lighting conditions vary across videos. Therefore, we transform images to the Hue-Saturation-Value (HSV) colour model (e.g. [10]). Pixels in HSV space are purified in colour by setting all saturation values to the maximum.

Transforming back to the RGB colour space yields a frame where searching for yellow pixels becomes a matter of simply choosing pixels with only a red-green component and no blue component. Denote (*x, y, z*) as the modified RGB image. The binary mask for yellow pixels, *M*_*yellow*_, is defined as,

(2)

The second binary mask selects pixels of low intensity from the original RGB frame, *f*(*x, y, z*), to localize the position of the black visor. This second mask is necessary since there are typically some yellow pixels not attributable to the target markers (e.g. window blinds or yellow-colored clothing). The yellow pixels of interest are those overlaying a dark background. Low intensity pixels are found by first converting each RGB frame, *f*(*x, y, z*), to grayscale, *g*(*x, y*). The grayscale image is then normalized and thresholded to select pixels less than 10% of the maximum intensity in the current frame. Let *g'*(*x, y*) denote the normalized grayscale image. The first form of the *N *× *M *binary mask for visor pixels, *M*_*b*1 _(*x, y*), is then:

(3)

Next, a morphological closing operation is applied to *M*_*b*1 _(*x, y*) to close the "holes" present in the visor area, especially the locations of the yellow markers that did not pass the filter for dark pixels:

(4)

where *J *is an empirically determined 7 × 7 structuring element consisting of a matrix of ones, •, ⊖ and ⊕ denotes image closing, erosion and dilation, respectively. A complete review of these operations is beyond the scope of this paper, and the reader should refer to [11] for more details.

The final result is a binary mask, *M *(*x, y*), that zeroes the majority of background pixels extraneous to the visor area:

(5)

The frame with nullified background, *h*(*x, y, z*), for each raw frame *f*(*x, y, z*) is then given by

(6)

**Figure 4:**
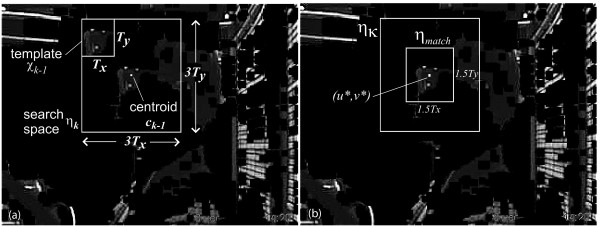
**Segmentation and template tracking steps**. Segmentation and template tracking: (a) example of a segmented frame, *h*_*k*_, showing the 3*T*_*x *_× 3*T*_*y *_search space, *η*_*k*_, centered around the centroid, *c*_*k*-1_, and template *χ*_*k*-1 _in its initial position; (b) identification of the best matched region *η*_*match *_centered around the point (u*, v*) yielding the highest cross-correlation in (7) within the search space *η*_*k *_in the *k*^*th *^frame.

Figure 4 depicts the result of segmentation of the *k*^*th *^frame, showing the 3 bright dots on the visor and the predominantly darkened pixels of the background.

#### Template Tracking

To reduce computational load, we define a *η*_*k*_(*x, y*) as a 3*T*_*x *_× 3*T*_*y *_subimage of the background-nullified frame, *h*_*k*_(*x, y, z*) as shown in Figure 4(a). This subimage *η*_*k*_(*x, y*) is centered around **c**_*k*-1_, the centroid of the three marker vertices from the previous frame and provides a reduced search space for template matching. We then calculate the normalized cross-correlation between the subimage *η*_*k *_and template *χ*_*k*-1 _at locations *γ*(*u, v*) in *η*_*k*_, in frame *k *≥ 1, according to:

(7)

where (*u, v*) range over the domain of *η*_*k*_,  is the mean intensity of the pixels in *η*_*k *_and  is the mean intensity of the pixels in the template. The summation is over all points (*x, y*) within the domain of the *T*_*x *_× *T*_*y *_template *χ*_*k*-1_. In other words, 1 + *u *≤ *x *≤ *T*_*x *_+ *u *and 1 + *v *≤ *y *≤ *T*_*y *_+ *v*. The point of highest correlation defines the region within the search space where the markers are most likely to be found. A subimage, *η*_*match*_(*x, y*) with dimensions 1.5*T*_*x *_× 1.5*T*_*y *_is defined around the point of highest correlation, (u*, v*) = arg max *γ*(*u, v*). At this point, *η*_*match*_(*x, y*) should contain collections of high intensity pixels contrasted against mostly dark background. The task of determining the actual coordinates of the markers still remains, as there may be multiple candidate pixels with high intensity in *η*_*match*_(*x, y*).

#### Determine Marker Coordinates

The non-zero pixels of the *η*_*match*_(*x, y*) image is first thresholded by Otsu's method [12] to select high intensity pixels. The Otsu algorithm seeks a threshold, T*, amongst the grayscale intensities such that interclass variance is maximized [12].

Using the Otsu threshold, T*, we derive a binary mask to select for the marker pixels:

(8)

The new search image, *ω*(*x, y*), is then created by applying our *M*_*otsu *_mask:

(9)

*ω*(*x, y*) is filtered with a sharpening filter to increase the intensity of the marker pixels:

(10)

with the following kernel being used for sharpening:

(11)

where Λ(*x, y*) is a 3 × 3 matrix with Λ(2, 2) = 1 and zero everywhere else, and Δ(*x, y*) is the Laplacian operator with the form and shape determined by the parameter *α *(we used *α *= 0.2) [11].

Next, a morphological reconstruction algorithm (e.g. [13]) is used to emphasize groups of high intensity pixels in Ω(*x, y*). In addition, a non-linear median filter is implemented to replace the value of each pixel with the median of the pixel values in a *q *× *r *area centered around that pixel (*q, r *∈ ℤ^+^). A 3 × 3 area is chosen because its size is smaller than the size of our markers and removed speckle noise that arose from the image sharpening operation. The median filtered image, Ω_*m*_(*x, y*), is then searched for candidate marker pixels.

We select candidate pixels by searching for local maxima in pixel intensity in Ω_*m*_(*x, y*) based on a four-neighbourhood approach. The set of candidate pixels, Γ, are then:

(12)

where

(13)

In the *k*^*th *^frame, *k *> 1, candidate triads of vertices are selected by searching for a set of three vertices in Γ that define a triangle similar to that in *χ*_0_. This process begins by searching for a pair of vertices (call them **p**_**1 **_and **p**_**2**_) in Γ, in descending order of intensity, whose line segment length, ||**p**_**1**_**p**_**2**_||, matches any of the side lengths, *L*_*i*_, *i *= 1, 2, 3, found in *χ*_0_. Matches are defined as differences in lengths of no more than 3 pixels. From this pair, the final vertex, **p**_**3**_, is found by considering the distance between **p**_**1 **_and other pixels in Γ, searching for matches to another side length in *χ*_0 _different from the length of ||**p**_**1**_**p**_**2**_||. This would account for two of the three reference lengths derived from *χ*_0_. The final vertex, **p**_**3**_, is a match only if the length ||**p**_3_**p**_2_|| coincided with the remaining side in *χ*_0_.

Hence, the vertices {**p**_1_, **p**_2_, **p**_3_} form a triangle with all three sides roughly matching that of the initial template. The process above is repeated until all candidates triads of vertices have been found from the set of pixels Γ. The best matching triad is the one which forms a triangle with side lengths and angles most closely resembling the corresponding reference measurements {*L*_1_, *L*_2_, *L*_3_, Θ_1_, Θ_2_, Θ_3_} derived from *χ*_0_. Simultaneously, the triangle formed by the best triad ought to be oriented at an angle similar to that of the previous frame. Thus, we define the following objective function, Ξ(*P*):

(14)

where the {*l*_*i*_, *θ*_*i*_} are the calculated side lengths and angles of the candidate triad, {*L*_*i*_, Θ_*i*_} are the corresponding side lengths and angles obtained from reference template *χ*_0_, *ϕ*_*k *_is the orientation of the candidate triad, *ϕ*_*k*-1 _is the orientation of the markers from the previous frame, and *I*_*i *_are the pixel intensities for each point in the candidate triad. The final term in (14) is included to favor triads containing high-intensity pixels.

The triad, P*, yielding the smallest Ξ value in (14), i.e.,

(15)

is selected to be the coordinates of the markers, which yield the corresponding neck angle, *ϕ*.

Following the angle measurement in the *k*^*th *^frame, the current template is replaced with the most recently derived triangle. This new template is deployed in the tracking of markers in the (*k *+ 1)^*th *^frame. The new template, *χ*_*k*_, has the same dimensions as the previous template (i.e., *T*_*x *_× *T*_*y*_) and is centred on **c**_**k**_, the centroid of the selected vertices. The template updates accommodate small rotations of the markers from frame to frame. Once a new template is defined, the algorithm iterates using the new template for locating the markers in the next frame.

### Data analysis

The image processing algorithm applied to each participant's video produced a time series of neck angles. However, erroneous neck angles resembling impulse noise arose due to the incorrect determination of markers coordinates. These erroneous points can skew neck angle averages if not filtered. Therefore, a series of pre-processing steps were applied to each neck angle time series to smooth the data and remove impulse noise. The steps included removal of zeros and points that are greater than two standard deviations from the signal mean; application of a non-linear median filter to remove large impulse noise; smoothing the final curve with an averaging filter and the removal of end data points.

Using the pre-processed time series, we calculated the average angle for each neutral and wet chin tuck position swallowing sequence. In addition, for the wet chin tuck swallows, we calculated the average maximum angles.

## Results and discussion

**Figure 5:**
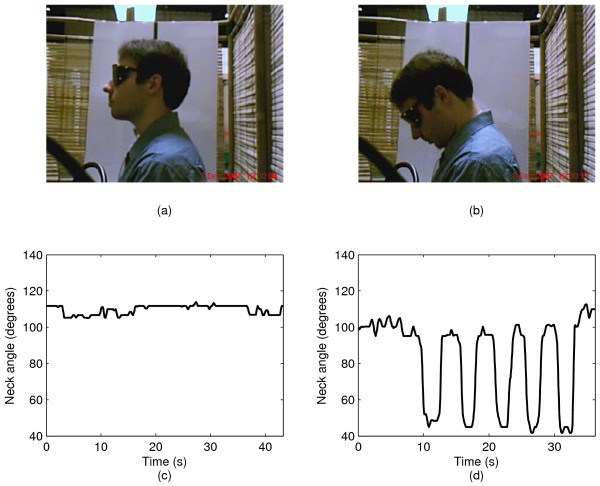
**A participant performing sample swallows**. A sequence of wet swallows in (a) neutral position and (b) chin-tuck position with corresponding neck angles, (c) and (d).

After the initial video screening, 230 participants were eliminated from further study, leaving a sample of 178 videos for subsequent analyses. Figure 5 depicts participant posture in wet swallows in (a) the neutral and (b) the chin-tuck positions. Corresponding neck angles are depicted in graphs (c) and (d). From the graphs, it is obvious that a participant nearly maintains a constant angle while performing wet swallows in the neutral position. In contrast, while performing wet chin tuck swallows, there are large angle variations due to the extensive head motion.

There were significant gender-based differences (Table 1) in neutral neck angles (Mann-Whitney test, *p *= 0.03) but no difference in the average (Mann-Whitney test, *p *= 0.05) and maximum (Mann-Whitney test, *p *= 0.13) angles for chin-tucks. Although the null hypothesis was rejected in the neutral head case, the *p*-value was very close to the critical value, indicating a weak rejection. Theoretically, there is little reason for the neutral neck angle of healthy participants to differ between genders and indeed this echoes literature [14,15]. Further, a study by Peng and Cooke on the reproducibility of head posture measurements found no inter-gender differences in neutral neck angle across a 15-year span [16]. The lack of inter-gender differences for chin-tuck neck angles is supported by studies finding no significant differences in cervical range of motion for neck flexion between genders [17-19].

**Table 1:** Neck angles between genders. Entries are mean ± standard deviations.

	**Overall**	**Male**	**Female**
Neutral position - avg. angle	97.4 ± 10.8	95.9 ± 11.9	100 ± 7.92
Chin tuck position - avg. angle	78.2 ± 9.81	77.3 ± 10.9	79.7 ± 7.52
Chin tuck position - max angle	52.5 ± 11.1	51.6 ± 11.6	54.1 ± 10.0

The results of the analysis with respect to age and BMI of participants are shown in Tables 2 and 3, respectively. Several observations are in order. Firstly, for wet chin tuck swallows, the extracted angles are not dependent on age (linear regression test, p > 0.55). This was surprising since we expected to see larger angles (less flexion) in the older age groups due to an age-related decrease in cervical range of motion [17,18]. However, these studies report only modest decreases in the range of motion - less than five degrees per decade - and any statistical significance would have been buried by the relatively high variance in our data. Even if chin-tuck neck angles do indeed carry an age-dependence, the clinical significance of such a trend may not be so pronounced since dysphagia predominantly occurs in the elderly [20,21]. The angles for neutral position wet swallows had a borderline dependence on age (*p *= 0.05), possibly due to the relatively large drop in the average neck angle for the oldest group. Tallgreen and Solow reported a similar age-related change in craniocervical posture [22]. Second, the extracted angles are not dependent on BMI (linear regression test, *p *> 0.71), which is in agreement with the study by Malmström et al. reporting BMI to have no significant influence on cervical flexion [23]. Overall, neck angles seem to be more consistent across individuals during wet chin tuck swallows than during neutral position wet swallows, which exhibit a mild age and gender association. Our data suggest that despite baseline differences (neck angle differences in the neutral position), healthy adults seem to perform the chin tuck task in a similar fashion, irrespective of age, BMI or gender. Variations in the effectiveness of chin tuck swallows may be unrelated to these anthropometric and demographic variables. Alternatively, the variations may not be adequately reflected in simple summary statistics (e.g., mean and maximum angle), and the entire waveform or its derivative (angular velocity) may need to be considered in future analyses.

**Table 2:** Neck angles across age groups. Entries are mean ± standard deviation.

	**18 ≤ Age < 35**	**35 ≤ Age < 45**	**45 ≤ Age < 55**	**55 ≤ Age < 65**
Neutral position - avg. angle	98.7 ± 8.82	98.3 ± 9.72	99.0 ± 7.70	93.9 ± 15.3
Chin tuck position - avg. angle	78.3 ± 8.99	78.1 ± 8.65	80.8 ± 11.1	76.0 ± 10.9
Chin tuck position - max. angle	52.8 ± 11.3	51.7 ± 10.6	53.3 ± 12.1	51.5 ± 11.0

**Table 3:** Angle variations with respect to BMI of participants.

	**BMI < 18.5**	**18.5 ≤ BMI < 25**	**25 ≤ BMI < 30**	**BMI ≥ 30**
Neutral position - avg. angle	97.5 ± 5.19	98.5 ± 10.2	95.9 ± 12.2	98.1 ± 10.6
Wet chin tuck position - avg. angle	78.1 ± 3.50	79.5 ± 10.9	76.0 ± 9.12	79.2 ± 9.30
Wet chin tuck position - max. angle	49.1 ± 4.47	53.1 ± 13.4	51.5 ± 9.24	52.5 ± 10.0

## Remarks

Minor variations in luminance did not affect the performance of our algorithm, since normalized cross-correlation coefficients used for template tracking are generally resistant to small changes in lighting [24]. In addition, the segmentation phase relied on the contrast between the dark visor and bright yellow dots. Minor changes in illumination may distort the intensity of the markers but they remain well-contrasted against the dark background of the visor. Furthermore, the lighting conditions for each data collection station provided relatively constant illumination within each video.

Neck circumference, body fat percentage and jaw length were found to be correlated with BMI (Pearson's *r *= 0.78, *r *= 0.73 and *r *= 0.25, respectively with *p *≈ 0) and were omitted from further analysis. In the present study, we have assumed minimal movement outside of the sagittal plane. While this is a reasonable assumption given the strict protocol, out-of-plane movement would have distorted the estimated neck angles. Our limited capture rate meant that very rapid chin tucks caused blurring of the markers, precluding accurate angle estimation. With some female participants with long hair, the markers were occluded during the chin tuck procedures. Future protocol should require loose, lengthy hair to be tied up. To mitigate these negative effects, we eliminated all participants who exhibited out-of-plane movement, marker blurring or marker occlusion from visual inspection of the recorded videos. This reduced the number of usable videos and hence weakened the strength of our findings. Nonetheless, our final results are based on a sample of 178 participants, which is still considered sizable for the analyses reported herein. We have only considered simple parametrizations (e.g., mean and maximum) of the neck angle waveform and other characterizations may be more closely associated with age, gender and BMI. Finally, we have only considered able-bodied individuals. The associations with anthropometric and demographic variables may be more pronounced in pathological populations.

## Conclusion

In this paper, head position during neutral and chin tuck position swallowing tasks has been studied. A template tracking algorithm for the automatic extraction of neck flexion angles from sagittal videos was proposed. Our analysis suggested that neck angles during chin tuck swallows have little association with age, BMI or gender in healthy adults, whereas there may be a weak gender and age differences in average neck angles during neutral position swallows.

## Competing interests

The authors declare that they have no competing interests.

## Authors' contributions

DH worked on the algorithm development and implementation, analyzed the data, and drafted the manuscript. ES proposed the main idea behind the algorithm, checked the method procedure, and participated in the data analysis and manuscript writing. CMS conceived the study, and helped to interpret the results and draft the manuscript. TC conceived the study, checked the method procedure, and participated in the data analysis and manuscript writing. All authors read and approved the final manuscript.
